# Causal Effects of Specific Gut Microbiota on Chronic Kidney Diseases and Renal Function—A Two-Sample Mendelian Randomization Study

**DOI:** 10.3390/nu15020360

**Published:** 2023-01-11

**Authors:** Ning Li, Yi Wang, Ping Wei, Yu Min, Manshu Yu, Guowei Zhou, Gui Yuan, Jinyi Sun, Huibo Dai, Enchao Zhou, Weiming He, Meixiao Sheng, Kun Gao, Min Zheng, Wei Sun, Dong Zhou, Lu Zhang

**Affiliations:** 1 Division of Nephrology, Affiliated Hospital of Nanjing University of Chinese Medicine, Jiangsu Province Hospital of Chinese Medicine, Nanjing 210029, China; 2 Department of Biotherapy and National Clinical Research Center, Sichuan University, Chengdu 610041, China; 3 Division of Nephrology, Department of Medicine, University of Connecticut, School of Medicine, Farmington, CT 06030, USA

**Keywords:** chronic kidney disease, gut microbiota, renal function, mendelian randomization

## Abstract

Background: Targeting the gut microbiota may become a new therapeutic to prevent and delay the progression of chronic kidney disease (CKD). Nonetheless, the causal relationship between specific intestinal flora and CKD is still unclear. Materials and Method: To identify genetically predicted microbiota, we used summary data from genome-wide association studies on gut microbiota in 18340 participants from 24 cohorts. Furthermore, we genetically predicted the causal relationship between 211 gut microbiotas and six phenotypes (outcomes) (CKD, estimated glomerular filtration rate (eGFR), urine albumin to creatinine ratio (UACR), dialysis, rapid progress to CKD, and rapid decline of eGFR). Four Mendelian randomization (MR) methods, including inverse variance weighted (IVW), MR-Egger, weighted median, and weighted mode were used to investigate the casual relationship between gut microbiotas and various outcomes. The result of IVW was deemed as the primary result. Then, Cochrane’s Q test, MR-Egger, and MR-PRESSO Global test were used to detect heterogeneity and pleiotropy. The leave-one method was used for testing the stability of MR results and Bonferroni-corrected was used to test the strength of the causal relationship between exposure and outcome. Results: Through the MR analysis of 211 microbiotas and six clinical phenotypes, a total of 36 intestinal microflora were found to be associated with various outcomes. Among them, Class Bacteroidia (=−0.005, 95% CI: −0.001 to −0.008, *p* = 0.002) has a strong causality with lower eGFR after the Bonferroni-corrected test, whereas phylum Actinobacteria (OR = 1.0009, 95%CI: 1.0003–1.0015, *p* = 0.0024) has a strong causal relationship with dialysis. The Cochrane’s Q test reveals that there is no significant heterogeneity between various single nucleotide polymorphisms. In addition, no significant level of pleiotropy was found according to MR-Egger and MR-PRESSO Global tests. Conclusions: Through the two-sample MR analysis, we identified the specific intestinal flora that has a causal relationship with the incidence and progression of CKD at the level of gene prediction, which may provide helpful biomarkers for early disease diagnosis and potential therapeutic targets for CKD.

## 1. Introduction

Chronic kidney disease (CKD) is a global health concern and affects nearly 700 million patients in the world [1]. With the progression of CKD, the renal function persistently declines, and renal replacement treatment is often necessary for patients going through the end-stage of the disease (ESKD) [2,3], which has brought a heavy burden on social and family medical expenditure [4]. In recent years, several new therapies have been found to delay the occurrence and progress of CKD [5,6]. Among them, hindering the progress of CKD by intervening in the gut–kidney axis is becoming a new research trend [7]. The gut microbiota, as the microorganisms regulating metabolism in the host intestine, also play an important role in the regulation of local and systemic immunity of the host [8,9]. CKD patients have certain micro-inflammatory states, and the disorder of intestinal flora will aggravate the imbalance of the immune system and the production of pro-inflammatory cytokines, leading to a systemic inflammatory response and further accelerating the progress of CKD and cardiovascular disease [10,11]. Emerging evidence shows that CKD patients are likely to have a certain degree of intestinal flora disorder [12,13]. As the disease progresses, the buildup of urea and other waste products causes intestinal dysbiosis and inflammation of the intestinal wall [14,15]. Some harmful metabolites (e.g., indoxyl sulfate, p-cresyl sulfate, indole-3 acetic acid, trimethylamine N-oxide (TMAO), and phenylacetylglutamine) produced by the disordered flora in turn will further aggravate the progress of CKD [16]. Therefore, the interactive dialogue based on the gut–kidney axis plays a primary role in the progression of CKD.

In recent years, the medical community worldwide has been targeting intestinal flora to regulate the incidence and progression of CKD [17,18]. Since there is an imbalance between harmful bacteria and probiotics in the intestinal tract of CKD patients, the alteration of intestinal flora may affect kidney injury and urinary toxin levels, a phenomenon that has been proved by previous animal model studies [13,19]. Several studies based on the stool of CKD patients for omics analysis also found that the alteration of gut bacteria was related to the severity of CKD [20,21]. Nevertheless, these studies are mainly based on observational cross-sectional analysis and cannot clarify the causal relationship. Mendelian randomization (MR) integrates summary data from genome-wide association studies (GWAS), minimizes the influence of confounding factors, and is often used to determine the possible correlation between exposure factors and outcomes [22]. Therefore, our study aims to investigate the causal relationship between specified gut microbiota, CKD, and renal function, which may provide guidance for developing helpful biomarkers for noninvasive diagnosis and potential therapeutic targets for CKD.

## 2. Method

### 2.1. Exposure and Outcome

This study considers a total of 211 intestinal microflora (131 genera, 35 families, 20 orders, 16 classes, and 9 phyla) with different attributes as exposure factors. There are six phenotypes (prespecified outcomes) included, and four of them are primarily from cross-sectional data: CKD (main outcome, defined as an estimated glomerular filtration rate (eGFR) of less than 60 mL/min/1.73 m^2^), eGFR (eGFR was calculated by using creatinine value according to CKD-EPI formula, and the results were analyzed by R software package (Nephro) [23]), urine albumin to creatinine ratio (UACR), and dialysis. To better reflect the dynamic impact of intestinal flora on CKD and renal function, we selected two additional endpoints in the form of cohort studies: rapid decline of kidney function (Rapid3) (the eGFR decreases by more than 3 mL/min/1.73 m^2^ per year), and rapid progress to CKD (CKDi25) (defined as the decrease of eGFR ≥ 25% of baseline accompanied by the progression from no CKD to CKD).

### 2.2. Data Source of Gut Microflora and Outcome

The full GWAS summary statistics towards the microbiota were derived primarily from a large-scale multi-ethnic GWAS meta-analysis (MiBioGen Consortium, www.mibiogen.org (accessed on 15 July 2022)) of 18,340 people from 24 cohorts which recorded 211 gut microbiota and 122,110 related single nucleotide polymorphis(SNPs) [24]. The summary statistics of instrument variables for CKD were derived from a meta-analysis by the Chronic Kidney Diseases Genetics Consortium (CKDGen Consortium) [25], which included 23 European ancestry cohorts (n = 480,698; 41,395 patients and 439,303 controls). In addition, the GWAS summary statistics of eGFR came from a meta-analysis [26], which included the data of the Chronic Kidney Disease Genetics (CKDGen) Consortium and UK Biobank (n = 1,201,909). The data of UACR were derived from a meta-analysis, which recorded the summary data of trans-ethnic (n = 564,257) and European-ancestry [27]. The summary statistics of Rapid3 (including 34,874 cases and 107,090 controls) and CKDi25 (encompassing 19,901 cases and 175,244 controls) were derived from a meta-analysis of 42 GWAS studies from the CKDGen Consortium and United Kingdom Biobank [28]. The GWAS summary statistics of dialysis were mainly extracted from UK Biobank (http://www.nealelab.is/uk-biobank, accessed on 15 July 2022). The datasets of CKD, eGFR, UACR, Rapid3, and CKDi25 are available at http://ckdgen.imbi.uni-freiburg.de/ (accessed on 15 July 2022). The profile of the included literature has been placed in the Supplement Table S2.

### 2.3. The Selection of Instrumental Variables

First, the instrumental variables selected for analysis need to be strongly correlated with exposure factors. To ensure sufficient instrumental variables screening, the SNPs with a *p*-value less than the locus-wide significance level (1 × 10^−5^) were selected. Furthermore, we excluded instrumental variables with *F* values (formula: (R^2^/(R^2^ − 1)) × ((N – K − 1)/K)) < 10 to ensure the strength of the association between instrumental variables and exposure factors. Secondly, the selected instrumental variables need to meet the independence test. To check the independence of these variables and the linkage disequilibrium effect, we set the linkage disequilibrium parameter (*R^2^*) of SNP to 0.001 and the genetic distance of 10,000 kb. Those with a MAF value of less than 0.01 are also excluded. Thirdly, since instrumental variables are not related to outcomes when the *p* value (outcome) of those variables was less than 0.05, they were excluded. The Phenoscanner [29] was used to check the possible confounding factors (i.e., hypertension, heart disease, diabetes, etc.) related to the instrumental variable, preventing such factors from interfering with the impact of exposure on outcomes. The above selection of instrumental variables ensures the reliability of our research results.

### 2.4. Mendelian Randomization Analysis

Inverse variance weighted (IVW), MR-Egger, weighted median, and weighted mode were used to investigate the causal relationship between exposure factors and outcome. IVW is a classic method that merges Wald ratio estimates of each instrumental variable in a meta-analysis, which is equivalent to implementing a weighted linear regression of the associations of the instrumental variables with the outcome. The intercept of the instrumental variables is constrained to zero. IVW is advantageous because it can obtain unbiased estimates of the status without horizontal pleiotropy. Differently, the MR-Egger method [30] is based on the assumption of InSIDE and mainly reflects the dose relationship between instrumental variables and outcomes, taking into account a certain level of pleiotropy. The weighted median method can reduce the occurrence of class 1 errors and allows some genetic variants to be invalid. When most instrumental variables with similar causal estimates are valid, the weighted mode approach is still credible even if some instrumental variables do not meet the requirements of the MR method for causal inference. If the outcomes of these methods are inconsistent, we prioritize to IVW as the main result. In order to guarantee that each IV was associated with the same effect allele, we harmonized the summary statistics, deleted SNPs with unclear strands (SNPs for A/T, C/G alleles), and aligned the summary statistics. The palindromic SNPs were removed to prevent the effect of alleles on the outcome of causality between gut microbiota taxa and CKD.

MR-Egger and MR Pleiotropy RESidual Sum and Outlier (MR-PRESSO) tests were used to test horizontal pleiotropy and outliers. MR-Egger was specifically applied to preliminarily identify the existence of horizontal pleiotropy. If the *p* value was greater than 0.05, it showed that there was no significant horizontal pleiotropy. Compared with MR-Egger, MR-PRESSO has higher accuracy and is useful in identifying horizontal pleiotropy and outliers [31]. Subsequently, Conchrane’s Q test was used for testing heterogeneity among instrument variables. The leave-one-out sensitivity analysis was used to test the outliers and the stability of the results. To obtain a more rigorous interpretation of the causal relationship, we also use the Bonferroni-corrected, according to the number of bacteria under each attribute (genera: 0.05/131 (3.81 × 10^−4^), families: 0.05/35 (1.4 × 10^−3^), orders: 0.05/20 (2.5 × 10^−3^), classes: 0.05/16 (3.1 × 10^−3^), and phyla: 0.05/9 (5.5 × 10^−3^). In addition, we conducted a sensitivity analysis using a fixed effects model to verify the reliability of the results. A reverse causality analysis is also conducted to examine the reverse causal association. The *p* value between 0.05 and the corrected value is considered to have a nominal causal effect. The STROBE-MR guideline was used to guide the design of this study [32]. The statistical analyses were performed using R software version 4.1.2 (https://www.rproject.org/, accessed on 15 July 2022).

## 3. Result

### 3.1. The Selection of Instrumental Variables

We screened the instrumental variables of 211 bacteria separately. A total of 14,587 instrumental variables achieved the locus-wide significance level (*p* < 1 × 10^−5^). After removing the linkage disequilibrium effect for specific flora, 3678 instrumental variables were retained. After eliminating the variables that are weakly related to exposure factors (*F* < 10, n = 17) and those that may be associated with confounding factors of outcomes (n = 30), a total of 3631 instrumental variables from 211 flora were finally included in the analysis. 

### 3.2. Two Samples MR Analysis

#### 3.2.1. CKD

**Figure 1:**
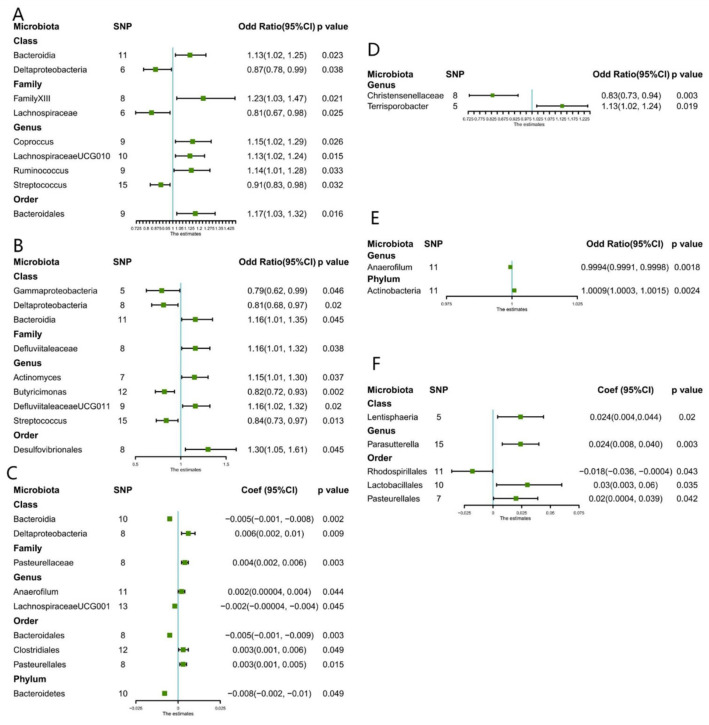
Forest plots of estimates identified with inverse variance weighted. (**A**) Chronic kidney disease, (**B**) CKDi25 (defined as the decrease in eGFR ≥ 25% of baseline accompanied by the progression from no CKD to CKD), (**C**) eGFR (estimated glomerular filtration rate), (**D**) Rapid3 (eGFR decreases by more than 3 mL/min/1.73 m^2^ per year), (**E**) Dialysis, (**F**) UACR (urine albumin to creatinine ratio).

This study identified nine causal relationships between the gut microbiota and the risk of developing CKD (Figure 1). A higher genetically predicted *Class Bacteroidia, Family FamilyXIII, Genus Coprococcus, Genus LachnospiraceaeUCG010, Gene Ruminococcus1, and Order Bacteroidales* were associated with a higher risk of CKD. Differently, *Class Deltaproteobacteria*, *Family Lachnospiraceae*, as well as *Genus Streptococcus* were associated with a lower risk. The MR-Egger and MR-PRESSO tests (Supplement Table S1) showed that there is no horizontal pleiotropy or outliers (*p* > 0.05). Furthermore, results from Cochrane’s Q test (Supplement Table S1) showed that no obvious heterogeneity was found in the selected SNPs (*p* > 0.05). Nonetheless, the leave-one-out method (Supplement Figure S9) demonstrated that some single SNPs might dominate the positive results of the above-exposed microbiota.

#### 3.2.2. eGFR

Nine causal relationships were found according to IVW analysis (Figure 1). A higher genetically predicted *Genus LachnospiraceaeUG001*, *Order Bacteroidales*, and *Phylum Bacteroidetes* were associated with a decrease in eGFR. While *Class Deltaproteobacteria*, *Family Pasteurellaceae*, *Genus Anaerofilum*, *Order Clostridiales*, and *Order Pasteurellales* were associated with an increase in eGFR. MR-Egger and MR-PRESSO tests (Supplement Table S1) showed that there is no horizontal pleiotropy or outliers (*p* > 0.05). Furthermore, no obvious heterogeneity was found according to results from Cochrane’s Q test (Supplement Table S1) (*p* > 0.05). In addition, the leave-one-out method (Supplement Figure S10) showed that except for *Class Bacteroidia*, *Class Deltaproteobacteria*, and *Family Pasteurellaceae*, some single SNPs might dominate the positive results.

#### 3.2.3. UACR

Between UACR, a causal correlation was found only in five microbiotas. A higher genetically predicted *Class Lentisphaeria*, *Genus Parasutterella*, *Order Pasteurellales*, and *Order Lactobacillales* were associated with an increase in proteinuria (Figure 1), while *Order Rhodospirillales* was associated with the decrease in proteinuria. No horizontal pleiotropy and outliers were seen according to the results of the MR-Egger and MR-PRESSO tests (Supplement Table S1) (*p* > 0.05). The outcomes from Cochrane’s Q test revealed no significant heterogeneity (Supplement Table S1) (*p* > 0.05). Finally, the leave-one-out method (Supplement Figure S11) pointed out that only *Genus Parasutterella* achieved stable results after excluding the SNP one by one.

#### 3.2.4. Dialysis

A higher genetically predicted *Genus Anaerofilum* was associated with a lower risk of dialysis, while the *Phylum Actinobacteria* was associated with a higher risk of dialysis. Furthermore, the outcomes from MR-Egger and MR-PRESSO tests (Supplement Table S1) confirmed that there is no horizontal pleiotropy (*p* > 0.05) and the outcomes from Cochrane’s Q test (Supplement Table S1) demonstrated that there is no obvious heterogeneity among the selected SNPs (*p* > 0.05). Also, no single outlier of SNP was identified after applying the leave-one-out method (Supplement Figure S12).

#### 3.2.5. CKDi25

Genetically predicted five microbiotas (Figure 1) were associated with an increased risk of CKDi25, including *Class Bacteroidia, Order Desulfovibrionales, Genus Actinomyces, Genus DefluviitaleaceaeUCG011, and Family Defluviitaleaceae.* Four microbiotas were associated with reduced risk of CKDi25, specifically *Genus Butyricimonas*, *Genus Streptococcus*, *Class Gammaproteobacteria*, and *Class Deltaproteobacteria*. MR-Egger and MR-PRESSO tests (Supplement Table S1) showed that there is no horizontal pleiotropy (*p* > 0.05) and, again, no obvious heterogeneity was found according to Cochrane’s Q test (Supplement Table S1) (*p* > 0.05). The leave-one-out method showed that except for *Genus Butyricimonas*, there may be some bias in other genetic predictions (Supplement Figure S13).

#### 3.2.6. Rapid3

A higher genetically predicted (Figure 1) *Genus Terrisporobacter* was associated with increased risk of Rapid3. Oppositely, *Genus Christensenellaceae* was associated with reduce risk of Rapid3. No significant heterogeneity and horizontal pleiotropy were found according to Cochrane’s Q, MR-Egger, and MR-PRESSO tests (Supplement Table S1). After removing the SNP one by one, the results remained stable (Supplement Figure S14).

### 3.3. Bonferroni-Corrected Test, Sensitivity Analysis, and Reverse Analysis

**Figure 2:**
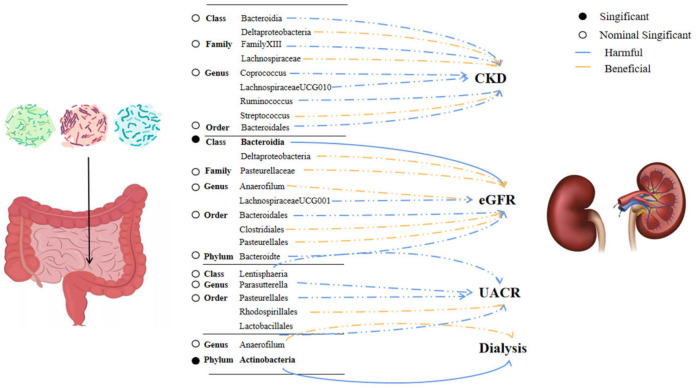
Significant and nominally significant links between kidney disease and intestinal bacteria. Abbreviation: CKD—chronic kidney disease, eGFR—estimated glomerular filtration rate, and UACR—urine albumin to creatinine ratio.

Results from the Bonferroni-corrected test revealed that a higher level of *Class Bacteroidia* retains a strong causal relationship with lower eGFR (*β* = −0.005, 95% CI: −0.001 to −0.008, *p* = 0.002), whereas a higher level of *Phylum Actinobacteria* retains a strong causal relationship with dialysis (OR = 1.0009, 95%CI: 1.0003–1.0015, *p* = 0.0024). Figure 2 showed the causal relationship between intestinal flora and various outcomes. After using the fixed effect model for the sensitivity analysis, the research results remained unchanged (Supplement Table S3). Reverse analysis (Supplement Table S4) demonstrates that CKD may lead to a higher rate of *Class Bacteroidia* (*p* = 0.03), but we did not observe a clear association for other microbiota. (*p* > 0.05).

## 4. Discussion

To the best of our knowledge, our study is the first large-scale comprehensive MR study that investigates the causality between intestinal microorganisms, CKD, and renal function at the level of gene prediction. Previous studies which investigated the association between intestinal flora and CKD were mainly conducted with animal models [12]. Several studies collected the feces of CKD patients, and the results obtained via omics analysis were based on the cross-sectional level, hence failing to explain the causal relationship between specific colonies causing CKD and renal function decline [20,21]. On the other hand, our study is based on new and large GWAS data and employs a gene prediction method to determine the relationship between specific flora and the occurrence and progression of CKD. Therefore, the results obtained are more authentic and have a reliable causal interpretation effect, which may provide some guidance for the future treatment of CKD by targeting specific gut microbiota.

In our study, we identified a total of 36 microflora that are associated with CKD and the progression of kidney function, and a strong causal relationship was identified in two of them. *Class Bacteroidia* (*β* = −0.005, 95% CI: −0.001 to −0.008, *p* = 0.0028) was associated with a lower level of eGFR, and *Phylum Actinobacteria* (OR = 1.009, 95%CI: 1.003–1.006, *p* = 0.002) was associated with dialysis. Reverse causality analysis revealed that CKD could also contribute to the increase of Bacteroidia, which suggests that the bacteria and CKD may interact with each other. In previous studies, Bacteroidia, as an obligate anaerobic gram-negative bacterium, has been reported many times to be related to the severity of CKD [33,34]. Bacteroidia have a gene that encodes a tryptophanase–tyrosine phenol-lyase that plays an important role in the production and accumulation of uremic toxins [34]. In a mouse nephrectomy CKD model, the accumulation of toxin levels was associated with the increased abundance of Bacteroides [35]. A study involving only Chinese individuals showed that, in patients with ESKD, the Prevotella dominant microbiota was decreased, and there was an accumulation of Bacteroidia [36]. We hypothesized that Bacteroidia caused the release of inflammatory mediators by producing corresponding toxins (e.g., indoxyl sulfate, TMAO, etc.) and accumulating in the blood circulation. These toxins and inflammatory mediators have been shown to be involved in the activation of the RAAS system [37], changes in the tissue microenvironment [38], and other pathways, which ultimately lead to the burden and damage of the kidney [39]. It is important to note that this buildup of toxins should result in various organ damage, not just the kidney. We can’t yet demonstrate the underlying mechanism because the goal of our research focuses on correlation analysis. Future studies on mechanism interpretation are required.

Our study based on genetic prediction found that there was a strong causal relationship between *Class Bacteroidia* and the decline of renal function. Interestingly, potential associations of *Class Bacteroidia* with CKD (OR: 1.13, 95%CI: 1.02–1.25, *p* = 0.02) and CKDi25(OR = 1.16, 95%CI: 1.00–1.35, *p* = 0.045) were also found, which seems to indicate that Bacteroides plays a crucial role in the occurrence and progression of CKD. Combined with the results of previous studies, targeted regulation of bacterial richness seems to be a new method to delay the progression of CKD. As a gram-positive prokaryotic microorganism, actinomycetes are still part of many discussions in the scholarly community regarding their association with CKD. Li et al. carried out 16S ribosomal DNA pyrosequencing on stool microbiota samples from patients with CKD and found that the abundance of actinomycetes was lower in the CKD group [40]; however, most related studies reveal opposite results. Al-Asmakh et al. demonstrated that the abundance of actinomycetes was increased in the intestinal tract of CKD rats [41]. Liu et al. revealed that the abundance of actinomycetes was increased in the fecal samples of CKD patients [42]. Vaziri et al. conducted a study with dialysis patients and reported that the abundance of actinomycetes increased in those with end-stage renal disease [12]. Our research confirmed that actinomycetes have a strong causal correlation with dialysis. Nonetheless, there is no relationship between CKD phenotypes and renal function. We are not sure whether this is due to the abundance of actinomycetes mainly increasing in more severe CKD stages (such as ESKD or those undergoing renal replacement treatment). Hence, we suggest that future studies focus on the association of actinomycetes with the severity of CKD.

It is worth noting that there is a possibility of a false negative for the Bonferroni-corrected test. Our study revealed that some microbiotas (e.g., *Class Deltaproteobacteria*, *Genus Streptococcus*, *Genus Anaerofilum*, and *Order Bacteroidales*) are commonly associated with various phenotypes. However, these correlations disappeared after the Bonferroni-corrected test. Similar to this, there are significantly less encouraging results when employing alternative MR-Egger approaches. We suppose that this may due to the crosstalk between intestinal and renal axes is often coordinated by multiple factors. The role of single microbiota in causing disease may not be as important as previously believed. Instead, a number of microorganisms could be coordinating and causing the disorders. It is commonly known that many bacteria participate in the regulation of kidney and intestinal pathophysiology [43]. These microorganisms, which have a nominal causality with multiple phenotypes, may also take part in the key dialogue between gut and kidney. Understanding the pathophysiology of the interactions between these microbiotas and kidney diseases can help us better comprehend the intricate mechanism of intestinal renal crosstalk and provide us with guidelines for the further development of targeted multi-flora drugs in the future.

Some mechanisms seem to preliminarily explain the relationship between intestinal flora and kidney disease. For instance, the gut microbial metabolites p-cresyl sulfate and indoxyl sulfate accumulation in the circulation results in increased intestinal permeability [15], and the systemic inflammation in blood vessels, endothelial dysfunction [44], insulin resistance [45], and activation of the renin–angiotensin–aldosterone system [46], may induce or aggravate the progress of CKD. Furthermore, it was previously verified that metabolic wastes and toxins in CKD patients further stimulate the disorder of the intestinal environment, forming a vicious cycle [10]. In addition, there is evidence that the harmful metabolites of disordered intestinal flora will stimulate the autonomic nervous system and then cause the excitation of sympathetic nerves, forming a vicious cycle between the brain–gut–kidney axis [47,48]. Complementarily, some studies proved that continuous sympathetic excitation would further promote the activation of the inflammatory system, inhibiting the repair of the kidney by pluripotent stem cells [45] and consequently causing common episodes of hypertension that can lead to abnormal renal perfusion [49]. Related studies also found that the transplantation of sterile fecal filtrate containing specific bacteria may degrade these metabolic wastes and improve renal injury and fibrosis in mice [20], which suggests that targeting specific intestinal flora may be a potential therapeutic target in CKD.

It is equally important to acknowledge the limitations of our study. First, the microbiome is an exposure phenotype limitedly explained by genotype, which mean that the robust calculation for Mendelian randomization statistical power would be too strict. Second, since the MR analysis is based on untestable assumption, further experimental and clinical validation study is crucial to test the clinical significance of microbial species. Third, although we set up two authors to check independently, there may still be some bias owing to subjective factors when using phenoscanner to remove the confounding influences of gene variables, so the interpretation of the research results still needs to be cautious. Last but not least, we used the alternative endpoint in our study, and there is no hard endpoint. The interpretation of the alternative endpoint is not as strong as the hard endpoint outcome, so it is necessary to carry out MR studies on compound hard endpoint in the future.

## 5. Conclusions

Through Mendelian randomization analysis of the causal relationship between 211 intestinal microflora and six phenotypes, we identified 34 nominal causalities and 2 strong causal associations. Among them, *Class Bacteroidia* is strongly associated with lower eGFR, while *Phylum Actinomycetes* are strongly associated with dialysis. Our research identifies specific microbiota using genetic prediction, which may provide helpful biomarkers for early disease diagnosis and potential therapeutic targets for CKD.

## Data Availability

The datasets used and analyzed in this study are available from the first author and corresponding author on reasonable request.

## Supplementary Materials

The following supporting information can be downloaded at: https://www.mdpi.com/article/10.3390/nu15020360/s1, Table S1. Mendelian randomized outliers and level pleiotropy test of exposure and outcome; Table S2. Summary presentation of included studies; Table S3. Sensitivity analysis for CKD (fix effect model); Table S4. Reverse causal analysis of CKD on gut microbiota; Figure S1. MR-Rgger analysis of causal effect between exposure microbiotas and various outcomes; Figure S2. Weight mean analysis of causal effect between exposure microbiotas and various outcomes; Figure S3. Scatter plots of significant and nominal significant estimates from genetically predicted microbiotas on CKD; Figure S4. Scatter plots of significant and nominal significant estimates from genetically predicted microbiotas on eGFR; Figure S5. Scatter plots of significant and nominal significant estimates from genetically predicted microbiotas on UACR; Figure S6. Scatter plots of significant and nominal significant estimates from genetically predicted microbiotas on dialysis; Figure S7. Scatter plots of significant and nominal significant estimates from genetically predicted microbiotas on CKDi25; Figure S8. Scatter plots of significant and nominal significant estimates from genetically predicted microbiotas on Rapid3; Figure S9. Leave-one-out plots of significant and nominal significant estimates from genetically predicted microbiotas on CKD; Figure S10. Leave-one-out plots of significant and nominal significant estimates from genetically predicted microbiotas on eGFR; Figure S11. Leave-one-out plots of significant and nominal significant estimates from genetically predicted microbiotas on UACR; Figure S12. Leave-one-out plots of significant and nominal significant estimates from genetically predicted microbiotas on dialysis; Figure S13. Leave-one-out plots of significant and nominal significant estimates from genetically predicted microbiotas on CKDi25; Figure S14. Leave-one-out plots of significant and nominal significant estimates from genetically predicted microbiotas on Rapid3; Figure S15. Funnel plots of significant and nominal significant estimates from genetically predicted microbiotas on CKD; Figure S16. Funnel plots of significant and nominal significant estimates from genetically predicted microbiotas on eGFR; Figure S17. Funnel plots of significant and nominal significant estimates from genetically predicted microbiotas on UACR; Figure S18. Funnel plots of significant and nominal significant estimates from genetically predicted microbiotas on dialysis; Figure S19. Funnel plots of significant and nominal significant estimates from genetically predicted microbiotas on CKDi25; Figure S20. Funnel plots of significant and nominal significant estimates from genetically predicted microbiotas on Rapid3.
