# Supplementary material for: Causal Effects of Specific Gut Microbiota on Chronic Kidney Diseases and Renal Function—A Two-Sample Mendelian Randomization Study
Source: Nutrients. 2023 Jan 11;15(2):360. doi: 10.3390/nu15020360 (PMC9863044; doi:10.3390/nu15020360)
Supplement: Supplementary file 1 [file nutrients-15-00360-s001.zip › Supplement file.pdf]

Supplementary Information

Causal effects of specific gut microbiota on CKD and renal function —— A two-sample mendelian randomization study

Ning Li[1]; Ping Wei[1]; Yu Min[2]; Manshu Yu[1]; Guowei Zhou[1]; Gui Yuan[3]; Jinyi Sun[1]; Huibo Dai[1]; Enchao Zhou[1*]; Weiming He[1]; Meixiao Sheng[1]; Kun Gao[1]; Min Zheng[1]; Wei Sun[1]; Dong Zhou[3]*; Lu ZHANG[1]*

Group	Exposure	Outcome	Cochran's Q-derived P value	MR-PRESSO-Global-test-derived P value	MR-Egger intercept-derived P value
**Class**	Bacteroidia	CKD	0.48	0.49	0.14
	Deltaproteobacteria	CKD	0.98	0.99	0.828
	Gammaproteobacteria	CKDi25	0.76	0.79	0.56
	Bacteroidia	CKDi25	0.64	0.64	0.32
	Deltaproteobacteria	CKDi25	0.22	0.27	0.58
	Bacteroidia	eGFR	0.67	0.69	0.33
	Deltaproteobacteria	eGFR	0.11	0.18	0.95
	Lentisphaeria	UACR	0.22	0.31	0.59
**Family**	FamilyXIII	CKD	0.58	0.60	0.347
	Lachnospiraceae	CKD	0.48	0.52	0.731
	Defluviitaleaceae	CKDi25	0.45	0.5	0.35
	Pasteurellaceae	eGFR	0.83	0.80	0.30
**Genus**	Coprococcus	CKD	0.81	0.83	0.613
	LachnospiraceaeUCG010	CKD	0.54	0.6	0.47
	Ruminococcus	CKD	0.53	0.53	0.81
	Streptococcus	CKD	0.77	0.78	0.59
	Actinomyces	CKDi25	0.76	0.77	0.90
	Butyricimonas	CKDi25	0.73	0.76	0.93
	DefluviitaleaceaeUCG011	CKDi25	0.51	0.56	0.35
	Streptococcus	CKDi25	0.44	0.47	0.59
	Anaerofilum	Dialysis	0.90	0.91	0.82
	Christensenellaceae	Rapid3	0.72	0.77	0.94
	Terrisporobacter	Rapid3	0.86	0.88	0.73
	Anaerofilum	eGFR	0.51	0.55	0.83

	Lachnospiraceae UCG001	eGFR	0.51	0.52	0.48
	Parasutterella	UACR	0.37	0.41	0.41
**Order**	Bacteroidales	CKD	0.35	0.4	0.26
	Desulfovibrionales	CKDi25	0.52	0.56	0.69
	Bacteroidales	eGFR	0.72	0.72	0.39
	Clostridiales	eGFR	0.27	0.30	0.95
	Pasteurellales	eGFR	0.37	0.34	0.66
	Pasteurellales	UACR	0.36	0.41	0.09
	Rhodospirillales	UACR	0.26	0.29	0.85
	Lactobacillales	UACR	0.09	0.13	0.96
**Phylum**	Actinobacteria	Dialysis	0.98	0.99	0.57
	Bacteroidetes	eGFR	0.29	0.29	0.13

Supplement Table S1. Mendelian randomized outliers and level pleiotropy test of exposure and outcome

Abbreviation: MR:Mendelian randomized, CKD: Chronic kidney disease, eGFR: estimated glomerular filtration rate, CKDi25: defined as the decrease of eGFR ≥ 25% of baseline accompanied by the progression from no CKD to CKD, Rapid3: eGFR decreases by more than 3 mL/min/1.73m2 per year, UACR: urine albumin to creatinine ratio.

Consortium	Author	Composition	Number	Research object
MiBioGen Consortium	Author Kurilshikov	24 cohorts	n=18340	211 gut microbiota
CKDGen Consortium	Wuttke M	23 cohort	41,395 patients and 439,303 controls	CKD
CKDGen Consortium	Stanzick KJ	GWAS summary statistics for CKDGen and UKB	n = 765,348 (CKDGen) and 436561 (UKB)	eGFR
CKDGen Consortium	Teumer A	54 GWAS summary statistics files	n=564,257	UACR
CKDGen Consortium	Mathias Gorski	42 genome-wide association studies	34,874 cases and 107,090 controls	Rapid3
CKDGen Consortium	Mathias Gorski	42 genome-wide association studies	19,901 cases, 175,244 controls	CKDi25

Supplement Table S2. Summary presentation of included studies

Exposure	nSNP	Odd ratio	95%lower	95%upper	P value
**Class**					
Bacteroidia	11	1.13	1.02	1.25	0.02
Deltaproteobacteria	6	0.87	0.77	0.98	0.04
**Family**					
FamilyXIII	8	1.19	1.01	1.39	0.038
Lachnospiraceae	6	0.84	0.72	0.98	0.026
**Genus**					
Coproccus	9	1.17	1.03	1.32	0.01
LachnospiraceaeUCG010	10	1.12	1.01	1.23	0.02
Ruminococcus	9	1.14	1.02	1.26	0.04
Streptococcus	15	0.93	0.86	0.99	0.03
**Order**					
Bacteroidiales	9	1.17	1.04	1.31	0.01

Supplement Table S3. Sensitivity analysis for CKD (fix effect model)

**Exposure**	**Outcome**	**β (95%CI)**	**p value**
	**Class**		
CKD	Bacteroidia	0.0003(0.0001, 0.0005)	0.03
	Deltaproteobacteria		
	**Family**		
CKD	FamilyXIII	0.0003(-0.0008, 0.0013)	0.56
	Lachnospiraceae	0.0008 (-0.0003,0.0012)	0.74
	**Genus**		
CKD	Coproccus	-0.0005(-0.0026, 0.0024)	0.83
CKD	LachnospiraceaeUCG010	-0.0002(-0.0004, 0.001)	0.73
CKD	Ruminococcus	0.0002(-0.0004, 0.0015)	0.61

CKD	Streptococcus	-0.0004(-0.002, 0.002)	0.45
	**Order**		
CKD	Bacteroidales	-0.001(-0.008, 0.001)	0.62

Supplement Table S4. Reverse causal analysis of CKD on gut microbiota

Supplement Figure S1

MR-Rgger analysis of causal effect between exposure microbiotas and various outcomes.

A) Categorical variable outcomes; B) Continuous variable outcomes

Abbreviation: MR:Mendelian randomized, CKD: Chronic kidney disease, eGFR: estimated glomerular filtration rate, CKDi25: defined as the decrease of eGFR ≥ 25% of baseline accompanied by the progression from no CKD to CKD, Rapid3: eGFR decreases by more than 3 mL/min/1.73m2 per year, UACR: urine albumin to creatinine ratio.

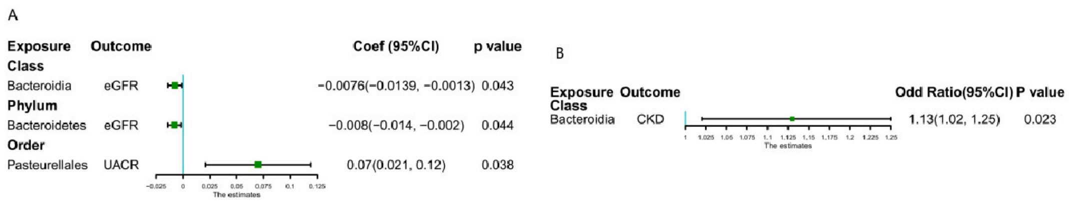

Supplement Figure S2

Weight mean analysis of causal effect between exposure microbiotas and various outcomes.

A) Categorical variable outcomes; B) Continuous variable outcomes

Abbreviation: MR:Mendelian randomized, CKD: Chronic kidney disease, eGFR: estimated glomerular filtration rate, CKDi25: defined as the decrease of eGFR ≥ 25% of baseline accompanied by the progression from no CKD to CKD, Rapid3: eGFR decreases by more than 3 mL/min/1.73m2 per year, UACR: urine albumin to

creatinine ratio.

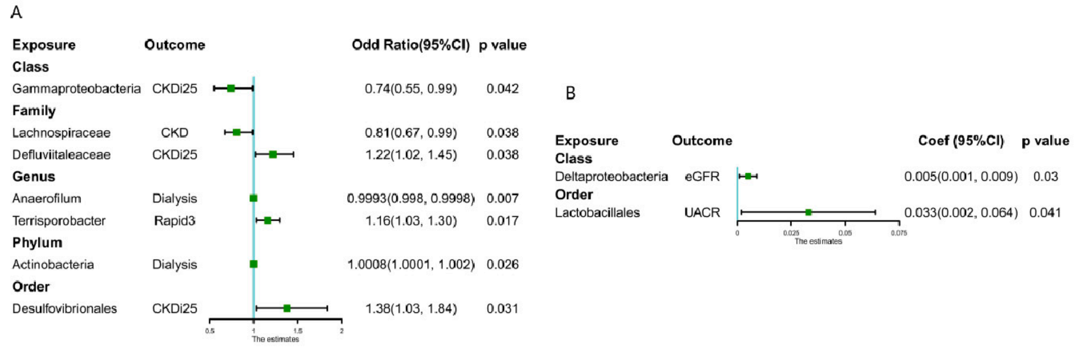

Supplement Figure S3

Scatter plots of significant and nominal significant estimates from genetically predicted microbiotas { (A) Class.Bacteroidia; (B) Genus.Coprococcus; (C) Class.Deltaproteobacteria; (D) Family.Lachnospiraceae; (E) Family.FamilyXIII ; (F) Genus.LachnospiraceaeUCG010; (G) Order.Bacteroidales; (H) Genus.Ruminococcus; (I) Genus.Streptococcus} on CKD

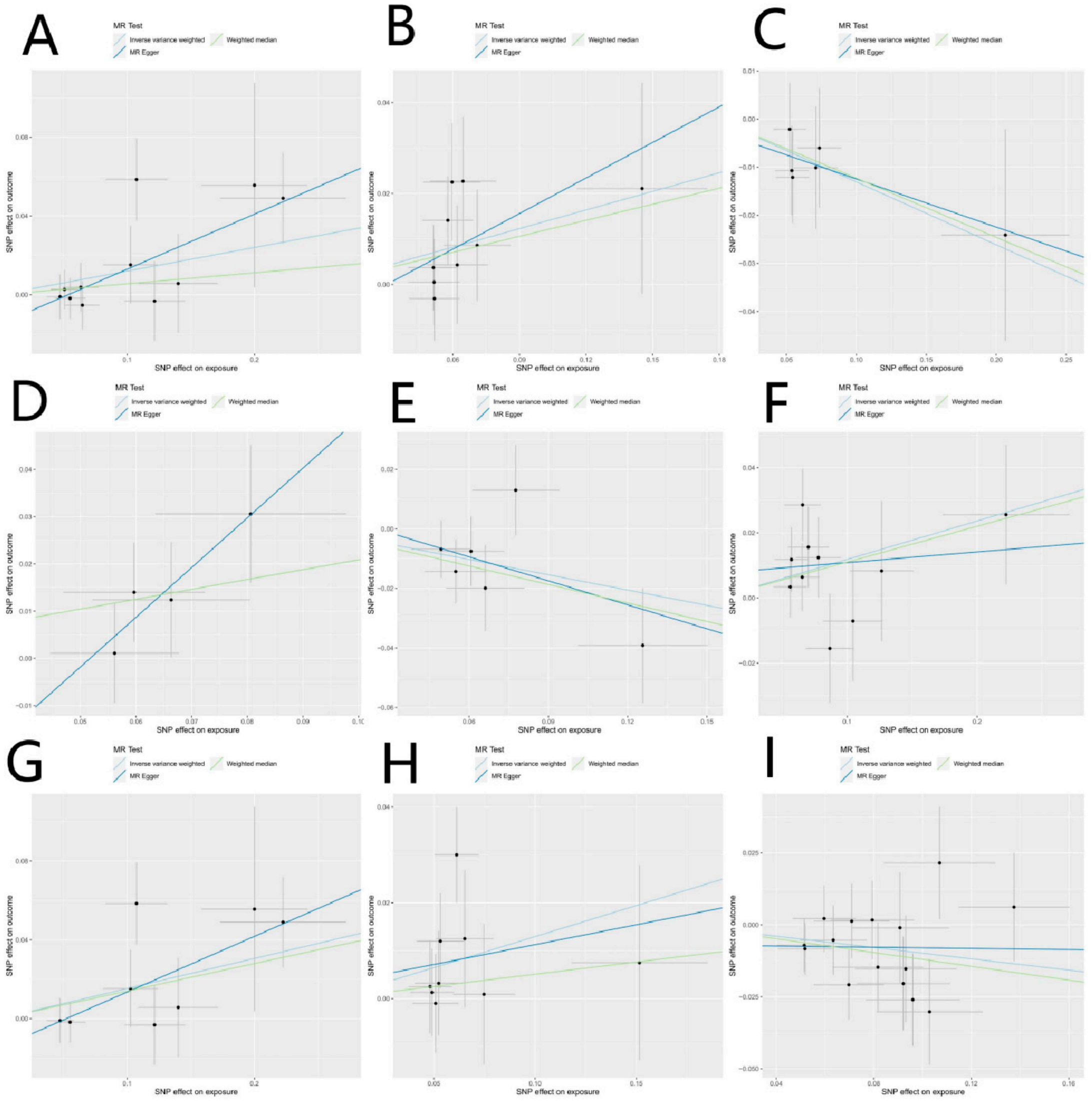

Supplement Figure S4

Scatter plots of significant and nominal significant estimates from genetically predicted microbiotas { (A) Class.Bacteroidia; (B) Class.Deltaproteobacteria; (C) Family.Pasteurellaceae; (D) Genus.Anaerofilum; (E) Genus.LachnospiraceaeUCG001 ; (F) Order.Bacteroidales; (G) Order.Clostridiales; (H) Order.Pasteurellales; (I) Phylum.Bacteroidetes} on eGFR

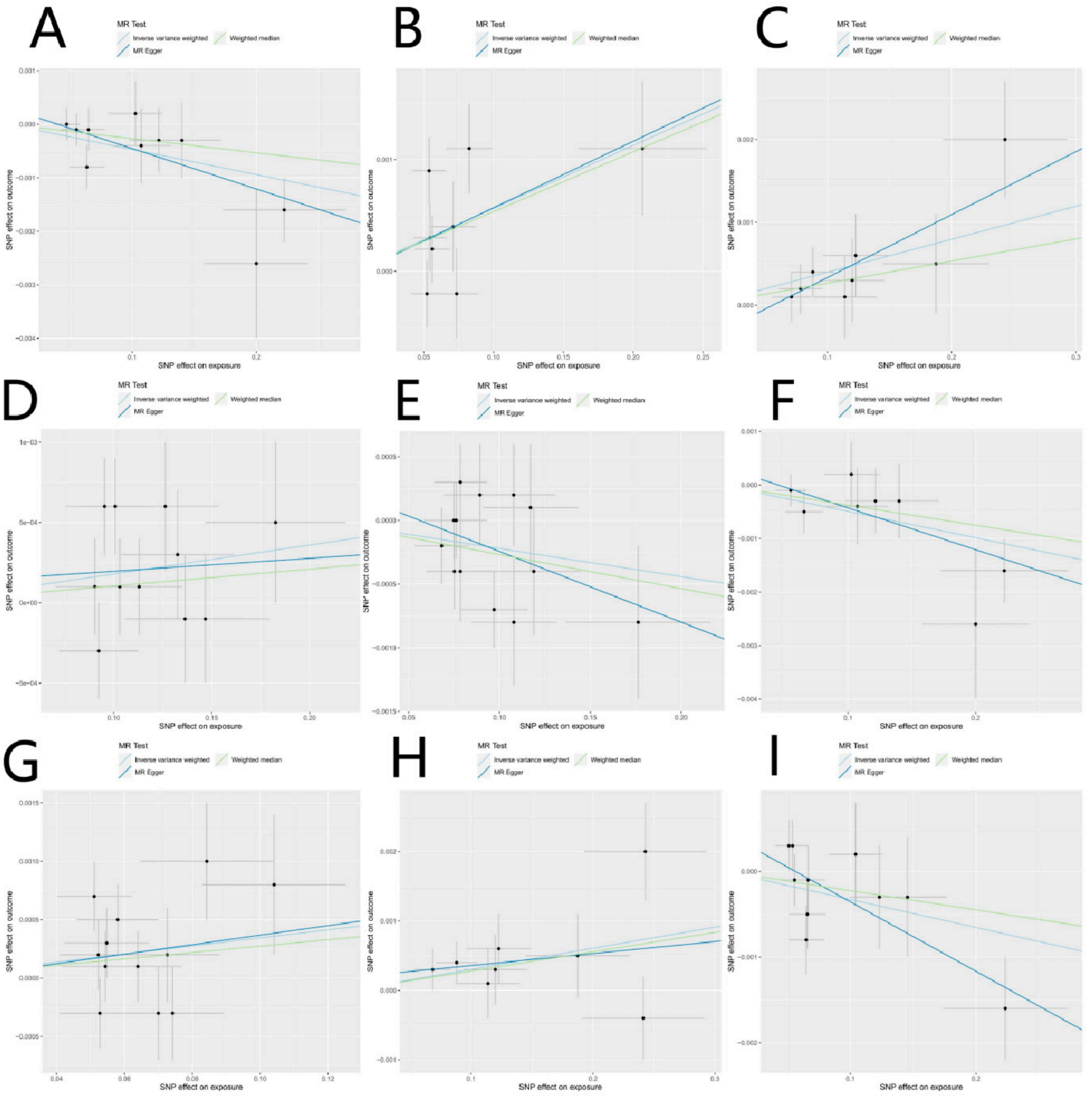

Supplement Figure S5

Scatter plots of significant and nominal significant estimates from genetically predicted microbiotas { (A) Class.Lentisphaeria; (B) Genus.Parasutterella; (C) Order.Lactobacillales; (D) Order.Pasteurellales; (E) Order.Rhodospirillales } on UACR

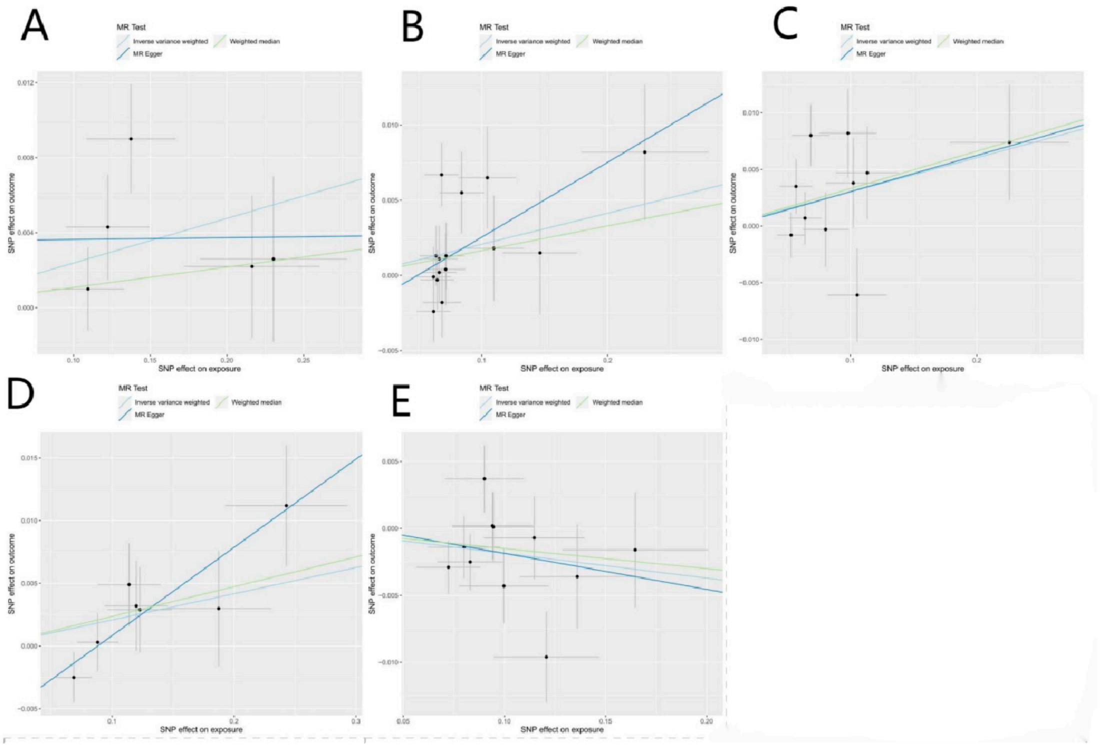

Supplement Figure S6

Scatter plots of significant and nominal significant estimates from genetically predicted microbiotas { (A) Phylum.Actinobacteria; (B) Genus.Anaerofilum } on Dialysis

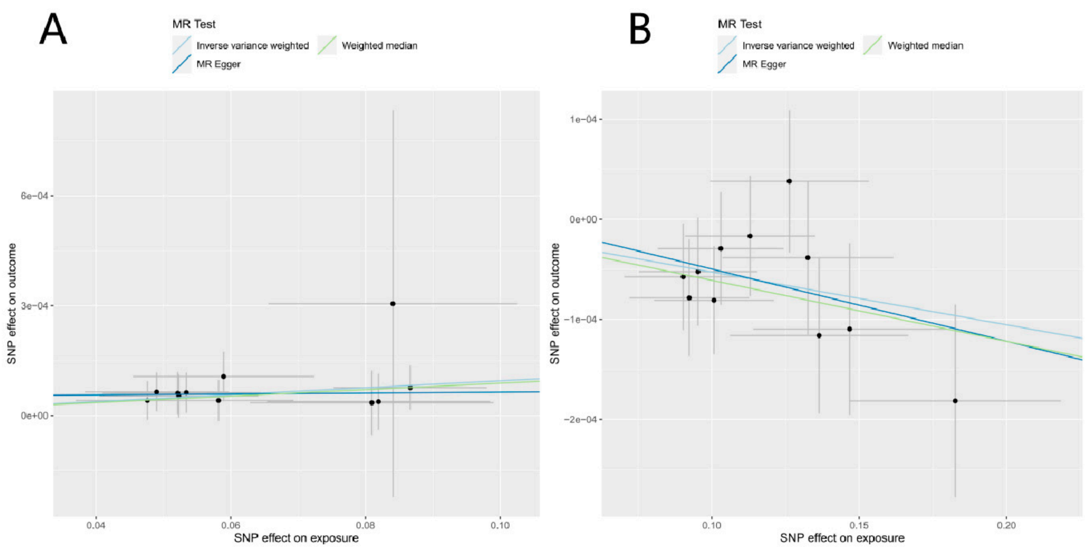

Supplement Figure S7

Scatter plots of significant and nominal significant estimates from genetically predicted microbiotas { (A) Class.Bacteroidia; (B) Class.Deltaproteobacteria; (C) Class.Gammaproteobacteria; (D) Family.Defluviitaleaceae; (E) Genus.Butyricimonas ; (F) Genus.Actinomyces; (G) Genus.DefluviitaleaceaeUCG011; (H) Genus.Streptococcus; (I) Order.Desulfovibrionales} on CKDi25

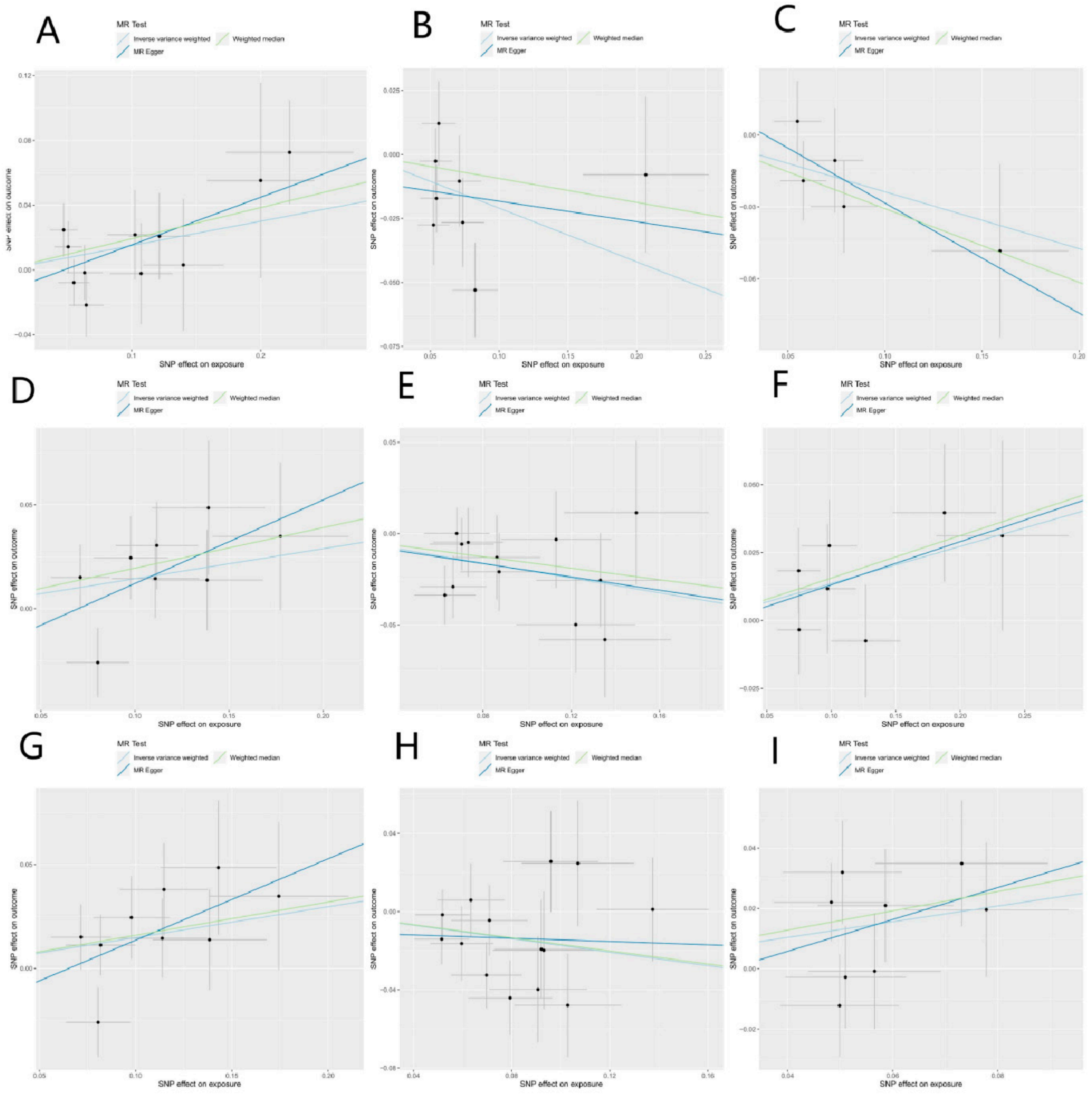

Supplement Figure S8

Scatter plots of significant and nominal significant estimates from genetically predicted microbiotas { (A) Genus.Christensenellaceae; (B) Genus.Terrisporobacter } on Rapid3

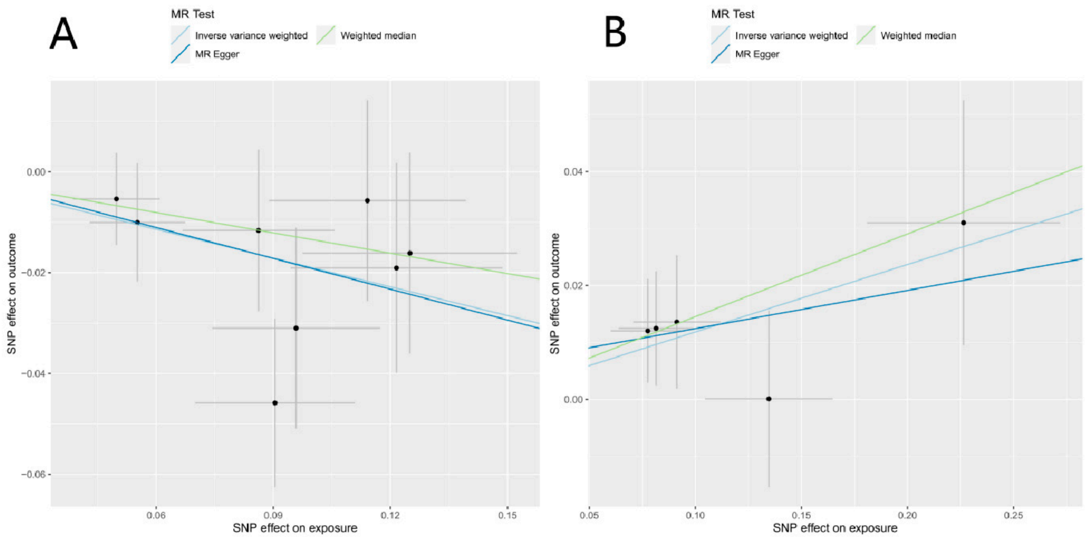

Supplement Figure S9

Leave-one-out plots of significant and nominal significant estimates from genetically predicted microbiotas { (A) Class.Bacteroidia; (B) Class.Deltaproteobacteria; (C) Family.Lachnospiraceae; (D) Family.FamilyXIII; (E) Genus.Streptococcus; (F) Genus.Coprococcus; (G) Genus.LachnospiraceaeUCG010; (H) Genus.Ruminococcus; (I) Order.Bacteroidales;} on CKD

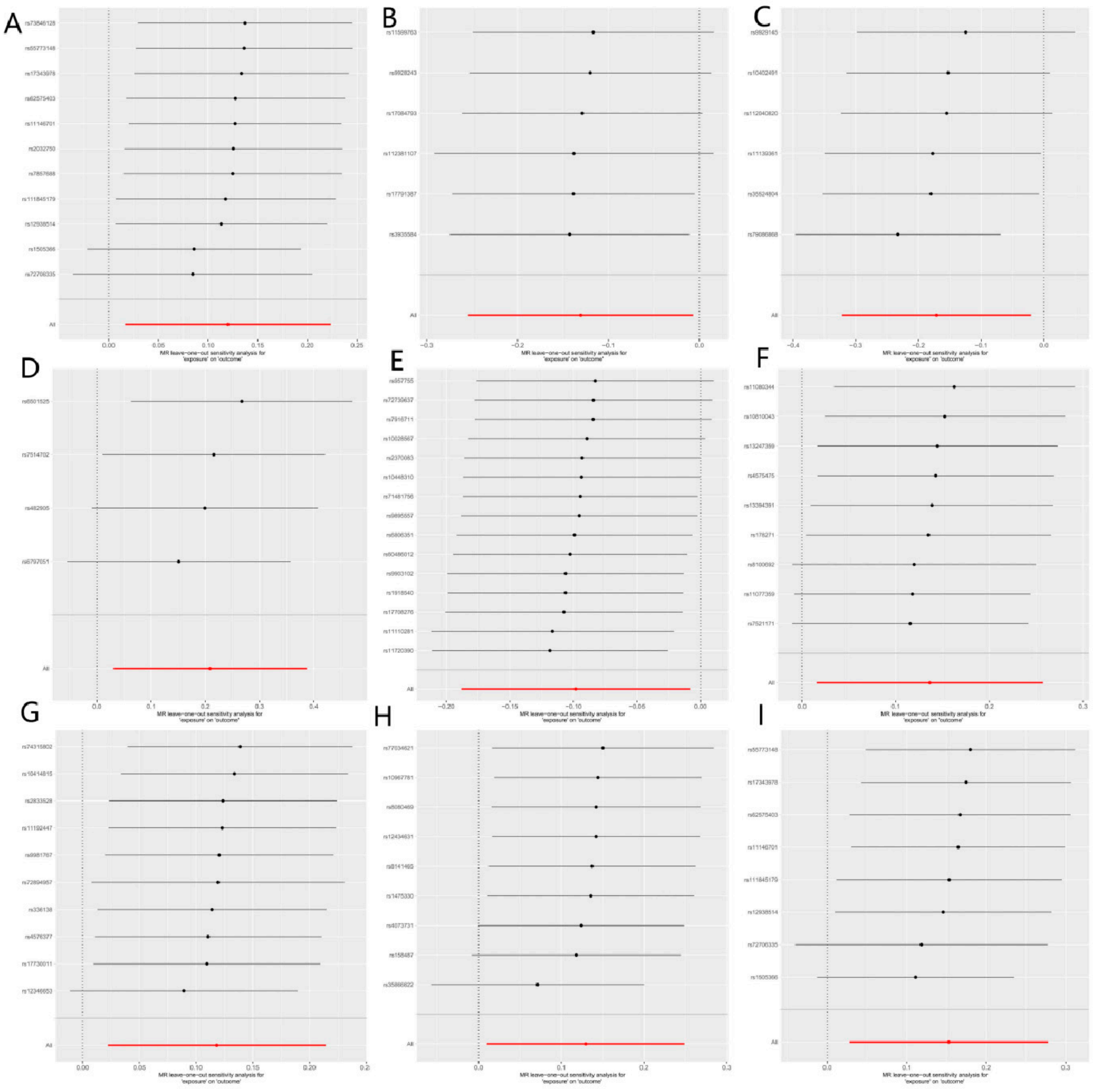

Supplement Figure S10

Leave-one-out plots of significant and nominal significant estimates from genetically predicted microbiotas { (A) Class.Bacteroidia; (B) Class.Deltaproteobacteria; (C) Family.Pasteurellaceae; (D) Genus.Anaerofilum; (E) Genus.LachnospiraceaeUCG001 ; (F) Order.Bacteroidales; (G)

Order.Clostridiales; (H) Order.Pasteurellales; (I) Phylum.Bacteroidetes} on eGFR

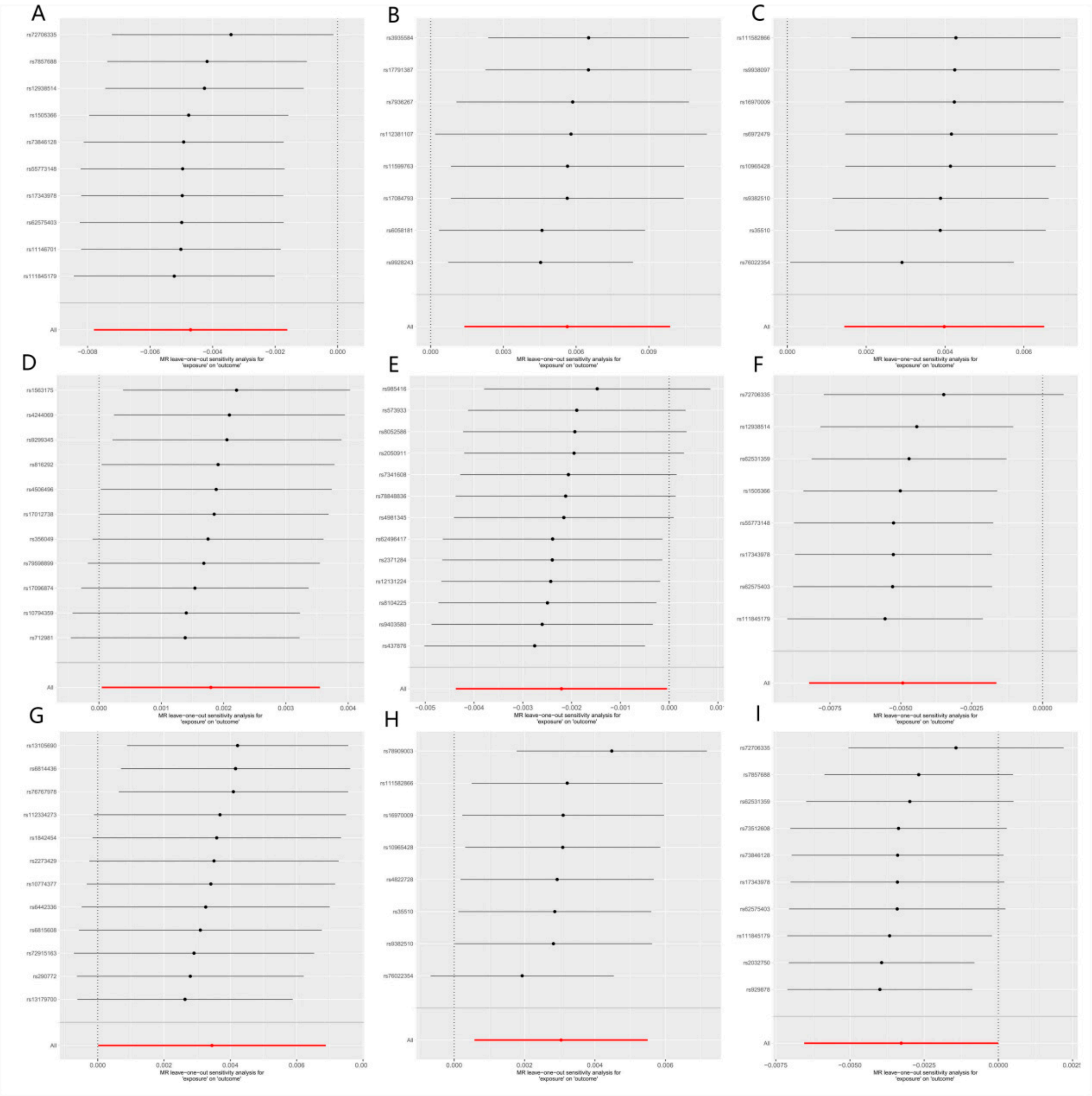

Supplement Figure S11

Leave-one-out plots of significant and nominal significant estimates from genetically predicted microbiotas { (A) Class.Lentisphaeria; (B) Genus.Parasutterella; (C) Order.Lactobacillales; (D) Order.Pasteurellales; (E) Order.Rhodospirillales } on UACR

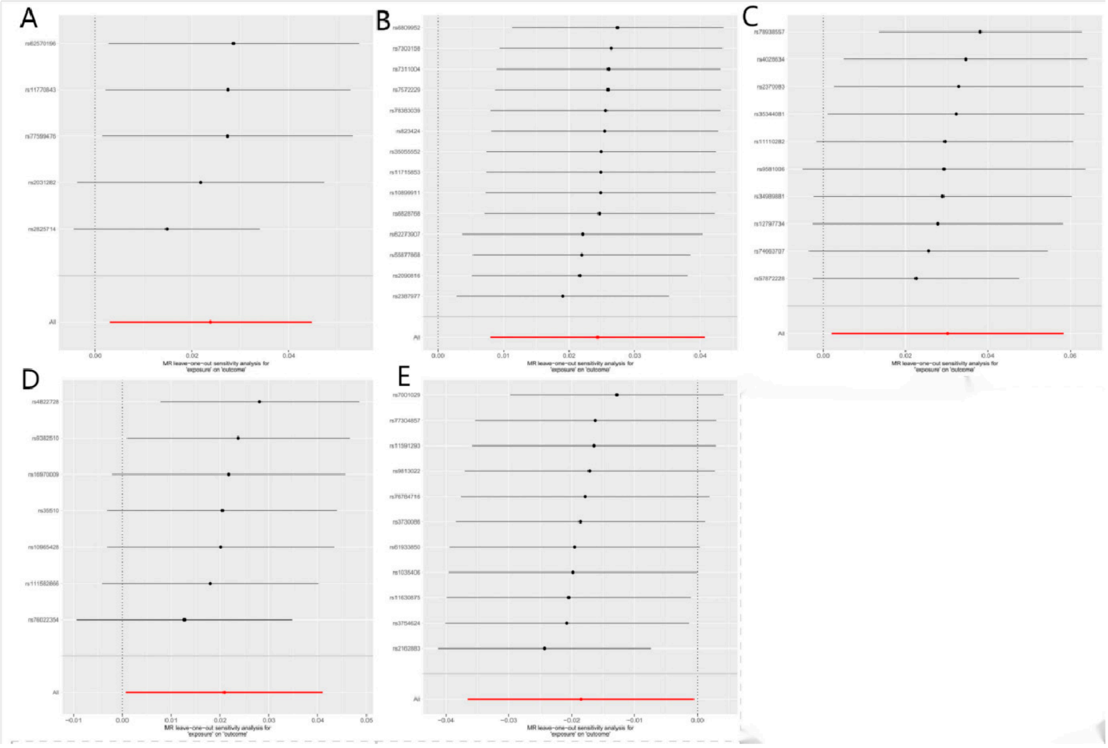

Supplement Figure S12

Leave-one-out plots of significant and nominal significant estimates from genetically predicted microbiotas { (A) Genus.Anaerofilum; (B) Phylum.Actinobacteria;   } on Dialysis

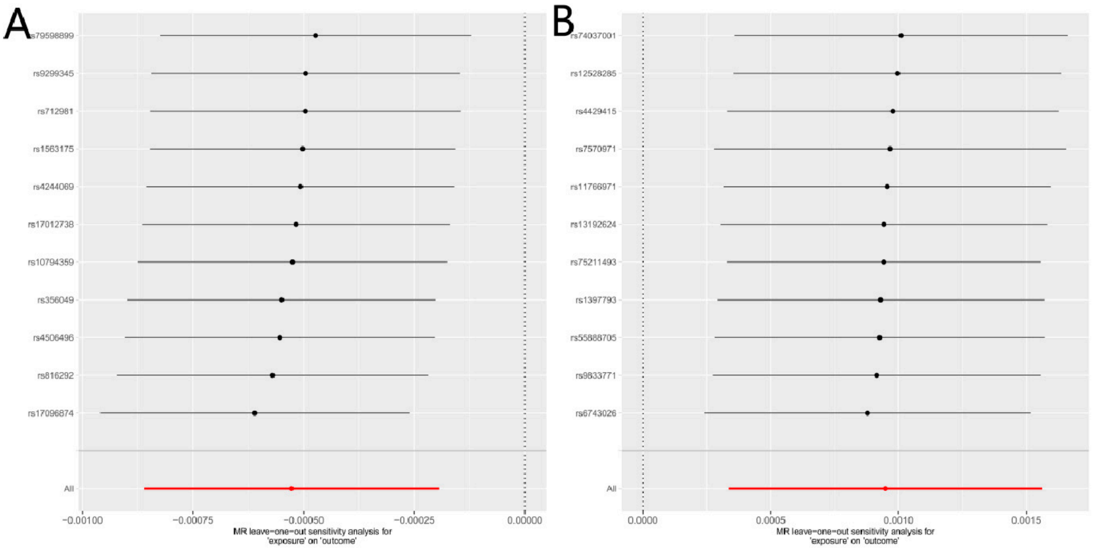

Supplement Figure S13

Leave-one-out plots of significant and nominal significant estimates from genetically predicted microbiotas { (A) Class.Bacteroidia; (B) Class.Deltaproteobacteria; (C) Class.Gammaproteobacteria; (D) Family.Defluviitaleaceae; (E) Genus.Actinomyces; ; (F) Genus.Butyricimonas (G) Genus.DefluviitaleaceaeUCG011; (H) Genus.Streptococcus; (I) Order.Desulfovibrionales} on CKDi25

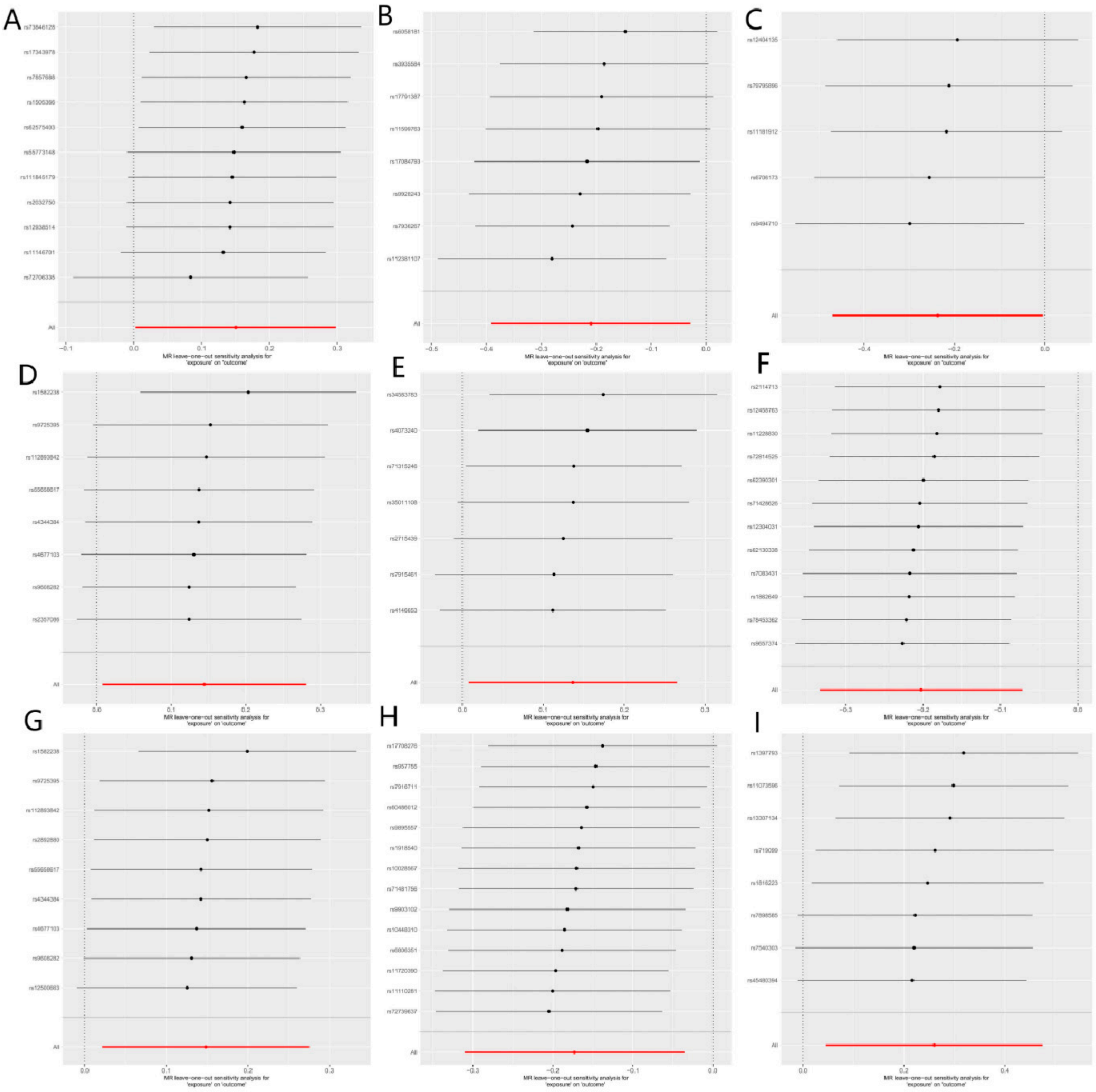

Supplement Figure S14

Leave-one-out plots of significant and nominal significant estimates from genetically predicted microbiotas { (A) Genus.Christensenellaceae; (B) Genus.Terrisporobacter } on Rapid3

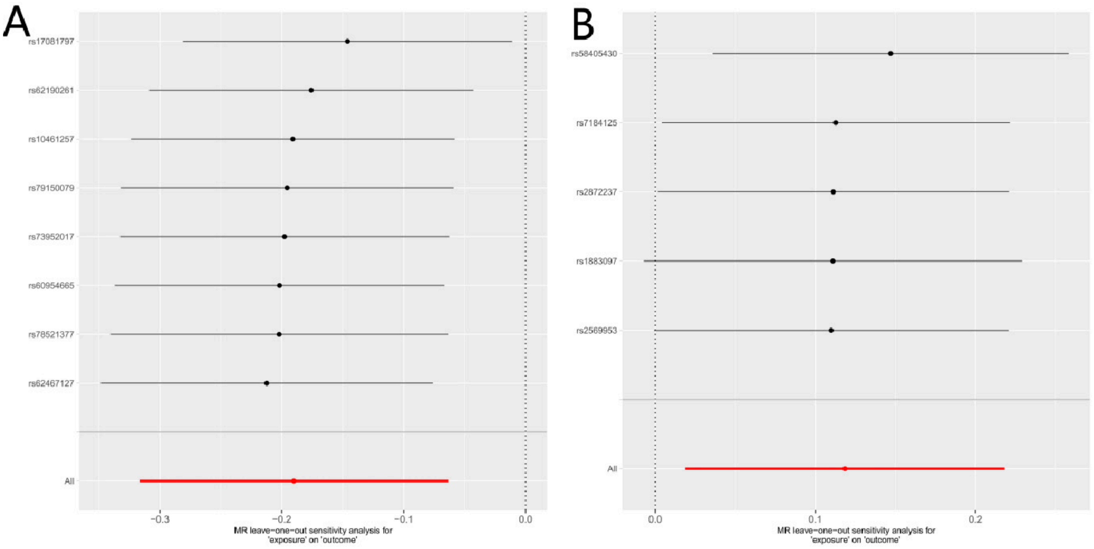

Supplement Figure S15

Funnel plots of significant and nominal significant estimates from genetically predicted microbiotas { (A) Class.Bacteroidia; (B) Class.Deltaproteobacteria; (C) Family.Lachnospiraceae; (D) Family.FamilyXIII; (E) Genus.Coprococcus; (F) Genus.LachnospiraceaeUCG010; (G) Genus.Ruminococcus; (H) Genus.Streptococcus; (I) Order.Bacteroidales;} on CKD

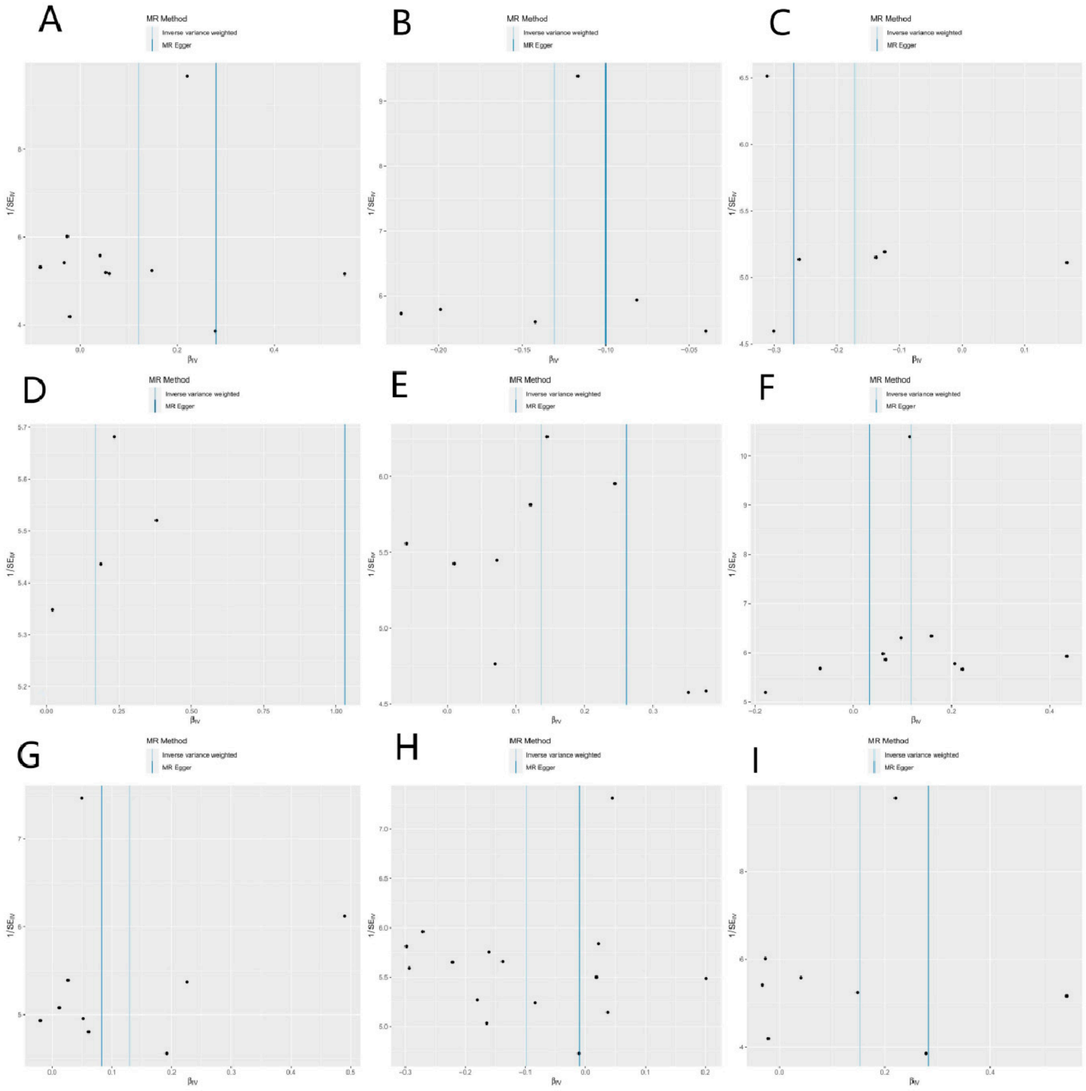

Supplement Figure S16

Funnel plots of significant and nominal significant estimates from genetically predicted microbiotas { (A) Class.Bacteroidia; (B) Class.Deltaproteobacteria; (C) Family.Pasteurellaceae; (D) Genus.Anaerofilum; (E) Genus.LachnospiraceaeUCG001 ; (F) Order.Bacteroidales; (G) Order.Clostridiales; (H) Order.Pasteurellales; (I) Phylum.Bacteroidetes} on eGFR

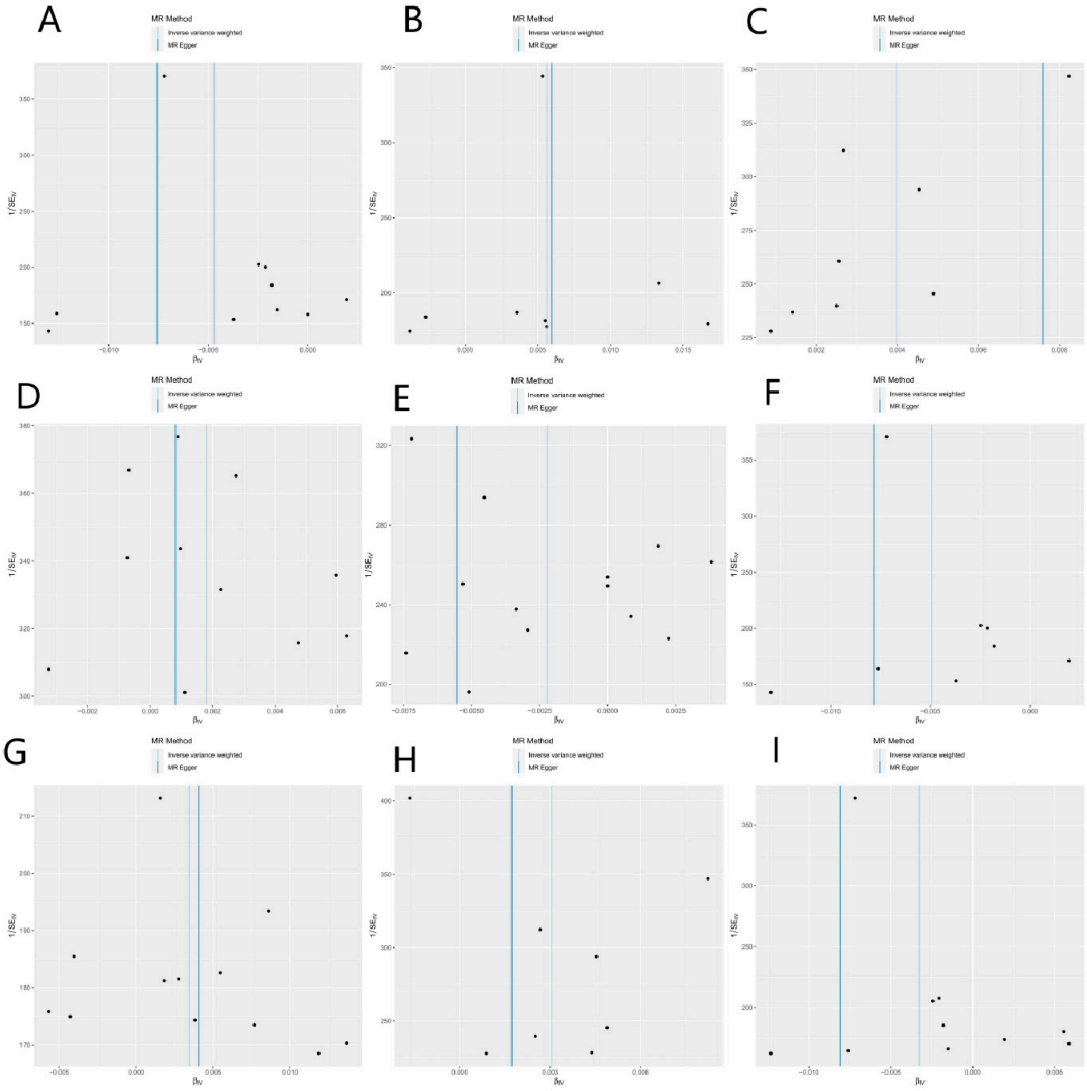

Supplement Figure S17

Funnel plots of significant and nominal significant estimates from genetically predicted microbiotas { (A) Class.Lentisphaeria; (B) Genus.Parasutterella; (C) Order.Lactobacillales; (D) Order.Pasteurellales; (E) Order.Rhodospirillales } on UACR

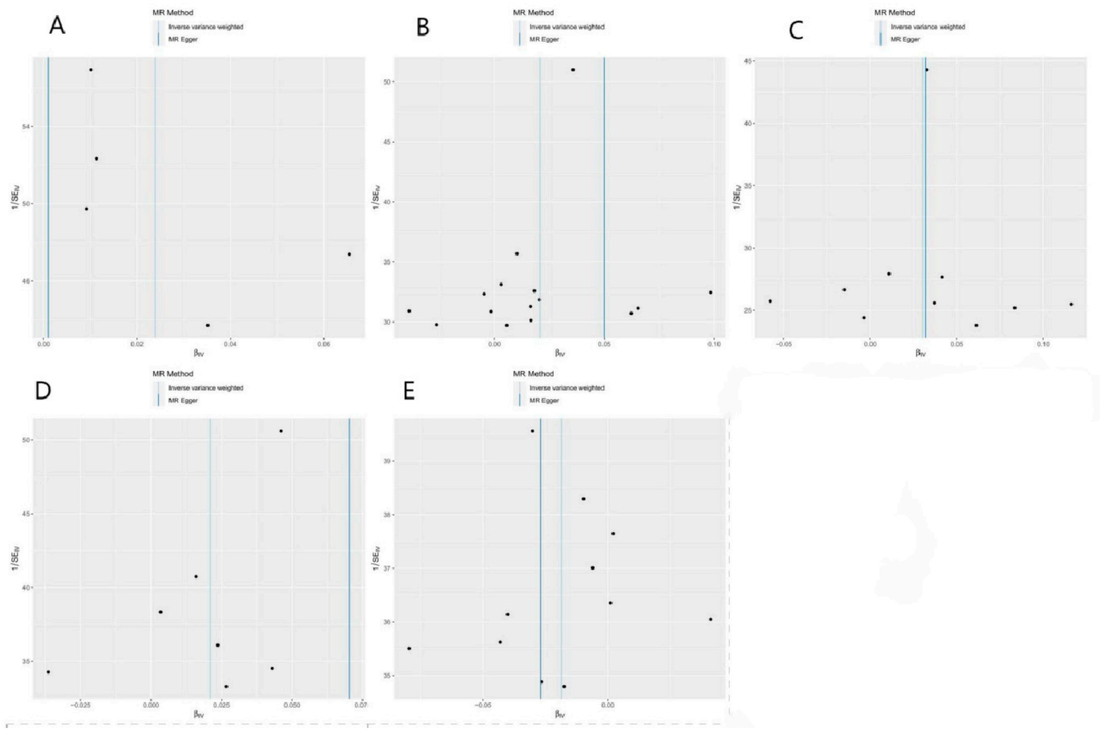

Supplement Figure S18

Funnel plots of significant and nominal significant estimates from genetically predicted microbiotas { (A) Genus.Anaerofilum; (B) Phylum.Actinobacteria; } on Dialysis

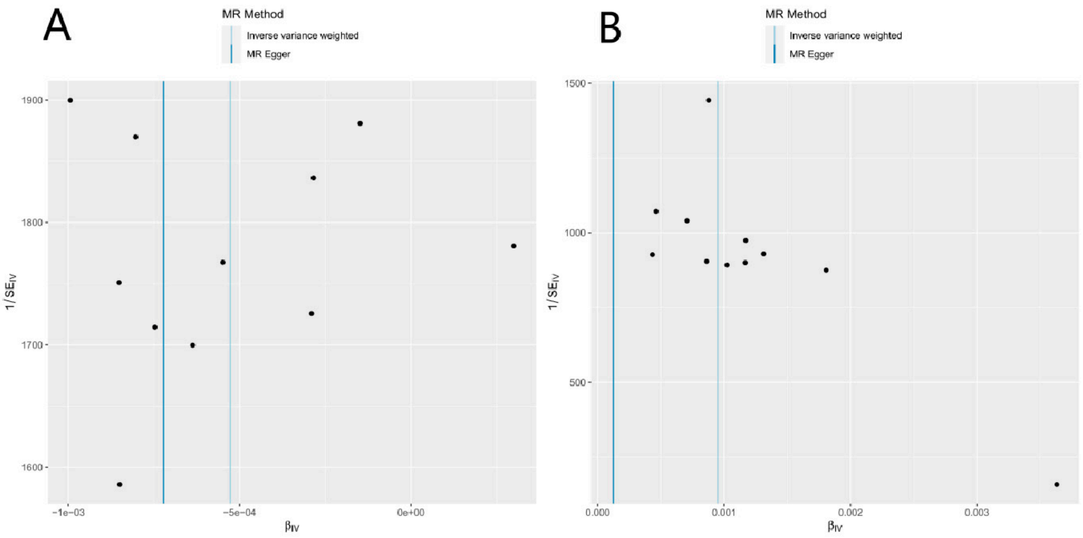

Supplement Figure S19

Funnel plots of significant and nominal significant estimates from genetically predicted microbiotas { (A) Class.Bacteroidia; (B) Class.Deltaproteobacteria; (C) Class.Gammaproteobacteria; (D) Family.Defluviitaleaceae; (E) Genus.Actinomyces; ; (F) Genus.Butyricimonas (G) Genus.DefluviitaleaceaeUCG011; (H) Genus.Streptococcus; (I)

Order.Desulfovibrionales} on CKDi25

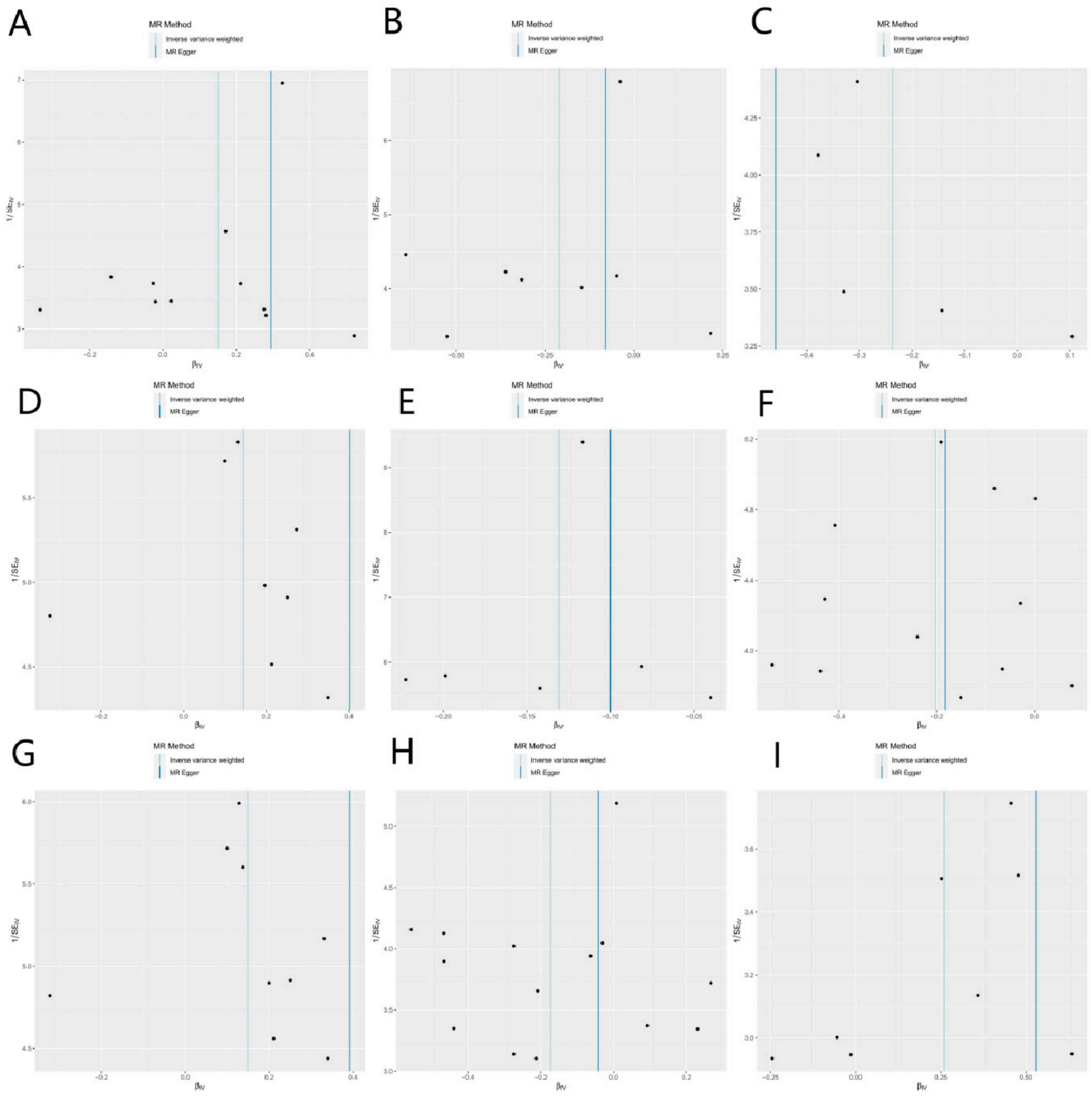

Supplement Figure S20

Funnel plots of significant and nominal significant estimates from genetically predicted microbiotas { (A) Genus.Christensenellaceae; (B) Genus.Terrisporobacter } on Rapid3

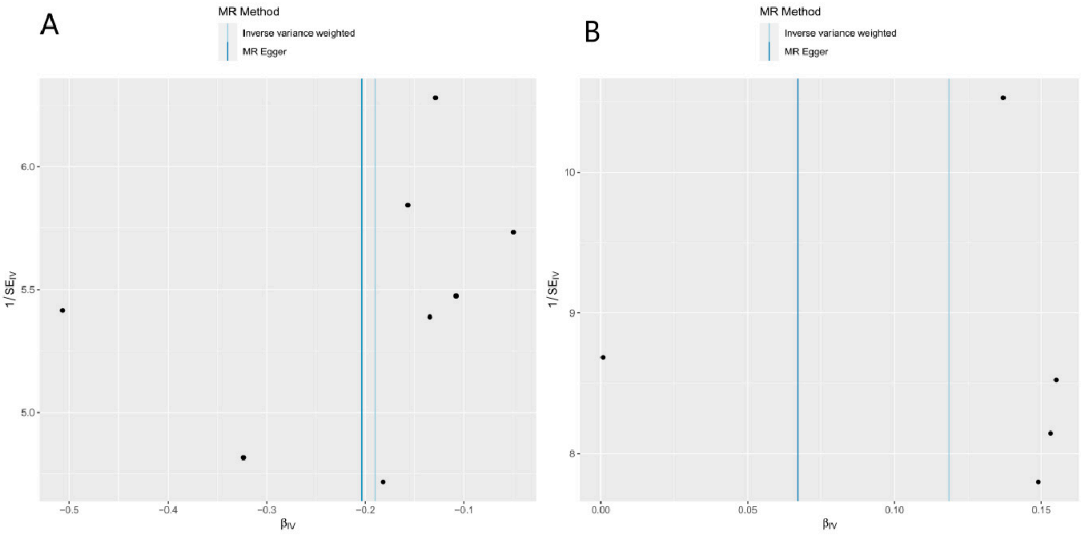
A
MR Method
Inverse variance weighted
MR Egger
6.0
5.5
5.0
1/SE_IV
−0.5
−0.4
−0.3
−0.2
−0.1
β_IV
B
MR Method
Inverse variance weighted
MR Egger
10
9
8
1/SE_IV
0.00
0.05
0.10
0.15
β_IV
